# Glycyrol Alleviates Acute Kidney Injury by Inhibiting Ferroptsis

**DOI:** 10.3390/ijms25052458

**Published:** 2024-02-20

**Authors:** Lixing Cao, Kai Han, Lihong Fan, Chong Zhao, Shutao Yin, Hongbo Hu

**Affiliations:** 1 College of Food Science and Nutritional Engineering, China Agricultural University, Beijing 100083, China; dolly1510505650@126.com (L.C.); hankai1122@cau.edu.cn (K.H.); zhaoch0206@cau.edu.cn (C.Z.); yinshutao@cau.edu.cn (S.Y.); 2 College of Veterinary Medicine, China Agricultural University, No. 2 Yuanmingyuan West Road, Haidian District, Beijing 100193, China; flh@cau.edu.cn

**Keywords:** glycyrol, ferroptosis, acute kidney injury, HO-1

## Abstract

Acute kidney injury (AKI) is a common clinical problem with high morbidity and mortality. The discovery of ferroptosis has provided novel insights into the mechanisms underlying AKI and paves the way for developing ferroptosis-based approaches to treat AKI. Glycyrol (GC) is a representative coumarin compound isolated from licorice that demonstrates various pharmacological activities. However, its potential for a protective effect against kidney injury remains unknown. We hypothesized that GC might be able to protect against AKI via suppression of ferroptosis. This hypothesis was tested in a cell-culture model of RSL3-induced nephrocyte ferroptosis and a mouse model of folic acid-induced AKI. The results showed that GC exerted a significant protective effect against nephrocyte ferroptosis in vitro and was effective against folic acid-induced AKI in vivo, where it was mechanistically associated with suppressing HO-1-mediated heme degradation. Collectively, the findings of the present study support the hypothesis that GC holds considerable potential to be developed as a novel agent for treating ferroptosis-related AKI.

## 1. Introduction

Ferroptosis is an iron-dependent and membrane-lipid peroxidation-mediated form of programmed or regulated cell death (RCD) that was first reported by Dixon et al. in 2012 [1]. The discovery of ferroptosis has provided novel insights into the pathogenesis of iron-dysregulation-related diseases. Ferroptosis is morphologically and biochemically distinct from other forms of RCD, such as apoptosis, necroptosis and unregulated necrosis. Morphologically, it is characterized by dramatic morphological changes in the mitochondria, such as shrinking with reduced or absent cristae, increased membrane density, and rupture of the outer membrane. Three key biochemical events have been found to be involved in ferroptosis induction, including an increase in the intracellular labile iron pool, iron-mediated membrane-lipid peroxidation and dysregulation of redox homeostasis [2]. The discovery of ferroptosis supports a mechanistic understanding the pathogenesis of various diseases and provides a strong rationale for developing ferroptosis-based approaches to manage ferroptosis-associated diseases.

Kidney diseases are the tenth leading cause of death worldwide, accounting for approximately 1.2 million deaths per year [3]. Kidney diseases are typically classified as either acute or chronic. Acute kidney injury (AKI) is defined by a sudden loss of excretory kidney function, characterized by elevated serum creatinine levels and reduced urinary output [4]. More than 20% of hospitalized patients and more than 50% of ICU patients suffer from AKI [5]. Due to its high incidence and mortality, AKI remains a major public-health problem. However, there are few effective treatments for AKI, and thus, novel approaches are needed for the effective management of AKI. A growing number of studies support the implication of ferroptosis in the pathogenesis of certain types of AKI, including ischemia-reperfusion kidney injury, AKI induced by nephrotoxic drugs (e.g., folic acid, cisplatin), glycerol-induced AKI, and sepsis-associated AKI [6,7,8]. Given the important contribution of ferroptosis to AKI, targeting ferroptosis is considered to be a plausible strategy by which to combat AKI.

Numerous studies have revealed that ferroptosis can be regulated by many phytochemicals, indicating the potential for using phytotherapy as an intervention to manage ferroptosis dysregulation-mediated AKI. Licorice, as a medicinal plant, has been widely used to treat various diseases in China and other Asian countries [9,10]. Over 400 components have been identified from licorice, mainly including flavonoids, coumarins, triterpenoids and stilbenoids [11,12]. Glycyrol (GC) is one of the major coumarin compounds in licorice and has been reported to exhibit multiple biological activities, including anti-cancer, anti-inflammation, anti-virus, hepatoprotective and neuroprotective activities [13,14]. However, less is known about its nephroprotective activity. We hypothesized that GC might be able to protect against kidney injury via suppression of ferroptosis. This hypothesis was tested in a cell-culture model of RSL3-induced ferroptosis of kidney cells and a mouse model of folic acid-induced renal injury.

## 2. Results

### 2.1. Glycyrol (GC) Exerts a Protective Effect on Nephrocyte Ferroptosis In Vitro

**Figure 1:**
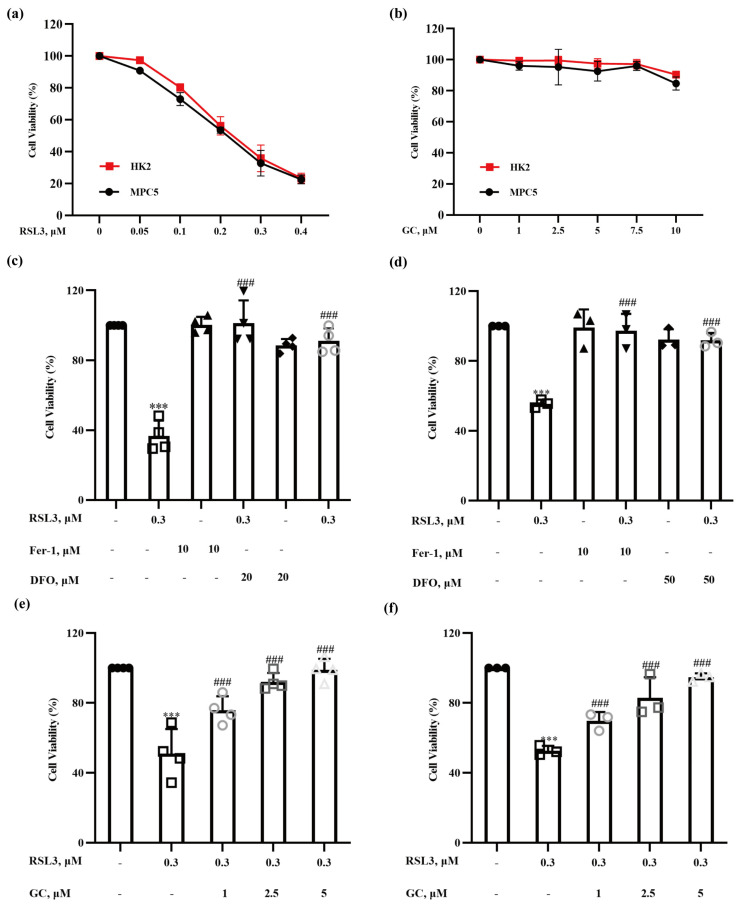
Glycyrol (GC) protected HK2 and MPC5 cells from ferroptosis. (**a**) HK2 and MPC5 cells were treated with the indicated concentrations of RSL3 for 18 h, and the changes in cell viability were determined by crystal violet staining; (**b**) HK2 and MPC5 cells were treated with the indicated concentrations of GC for 18 h, and the changes in cell viability were determined by crystal violet staining; (**c**,**d**) HK2 and MPC5 cells were treated with RSL3 (0.3 μM) in the absence or presence of different concentrations of ferrostatin-1 (Fer-1) and deferoxamine (DFO) for 18 h, and then cell viability was assayed; (**e**,**f**) HK2 and MPC5 cells were treated with RSL3 (0.3 μM) in the absence or presence of different concentrations of GC for 18 h, and then cell viability was assayed. Data are represented as the means ± SD of triplicate experiments. (*** *p* < 0.001 versus the control group. ### *p* < 0.001 versus the RSL3 group).

To investigate the protective effect of GC on nephrocyte ferroptosis, we first established a cell-culture model of ferroptosis induction in nephrocytes. HK2 human proximal tubular epithelial cells and MPC5 murine podocyte cells were treated with various concentrations of RSL3, a well-known inducer of ferroptosis that inhibits GPX4 activity [15], for 18 h, and cell viability was measured by crystal violet staining. As shown in Figure 1a, the viability of 2 types of cells decreased in a dose-dependent manner following the treatments. As expected, RSL3-induced reduction of cell viability was nearly abolished by ferrostatin-1 and DFO, two classical inhibitors of ferroptosis (Figure 1b,c), indicating that the reduction in cell viability by RSL3 occurs predominantly via induction of ferroptosis in these two types of kidney cell. To determine the non-toxic concentrations of GC, HK2 and MPC5, cells were exposed to GC at concentrations of 1, 2.5, 5, 7.5 and 10 μM for 18 h, and the changes in cell viability were evaluated by crystal violet staining; the results are shown in Figure 1d. GC at the concentrations used above did not induce significant cytotoxicity in either HK2 or MPC5 cells. We then examined the effect of GC at these non-toxic concentrations on RSL3-induced ferroptosis in HK2 and MPC5 cells. As shown in Figure 1e,f, GC at concentrations of 1, 2.5 and 5 μΜ prevented the RSL3-induced reduction in cell viability in a dose-dependent manner in these two cell lines tested. The results suggest that GC is able to protect kidney cells from RSL3-induced ferroptosis.

### 2.2. The Protective Effect of GC on Nephrocyte Ferroptosis Is Associated with Its Specific Structural Feature

**Figure 2:**
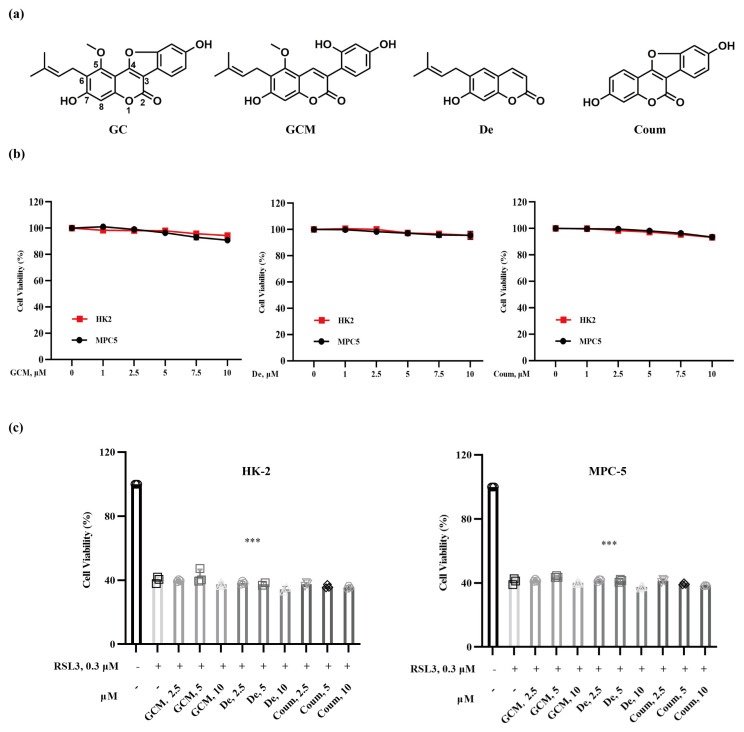
The structure–activity relationship of glycyrol in the context of its protective effect against ferroptosis. (**a**) Chemical structures of glycyrol (GC) and its analogues, glycycoumarin (GCM), demethylsuberosin (De) and coumestrol (Coum); (**b**) HK2 and MPC5 cells were treated with the indicated concentrations of GCM, De and Coum for 18 h, and the changes in cell viability were determined by crystal violet staining; (**c**) HK2 and MPC5 cells were treated with RSL3 (0.3 μM) in the absence or presence of different concentrations of GCM, De and Coum for 18 h, and then cell viability was assayed. Data are represented as the means ± SD of triplicate experiments. (*** *p* < 0.001 versus the control group).

Glycycoumarin (GCM), demethylsuberosin (De) and coumestrol (Coum) are structural analogues of GC (Figure 2a) that are basically composed of a benzene ring fused to α-pyrone ring. In comparison with GC structure, the furan ring at c-3/4 is open in GCM, the benzofuranyl group at c-3/4 is absent in De, and the isopentenyl group at c-6 is absent in Coum. To explore the structure–activity relationship of GC in the context of its activity against nephrocyte ferroptosis, we first evaluated the influence of GCM, De, or Coum alone on the viability of HK2 and MPC5 cells. As shown in Figure 2b, similar to GC, GCM, De and Coum at concentrations of 1, 2.5, 5, 7.5 and 10 μM did not cause significant cytotoxicity in either cell line tested. Next, we measured the influence of these compounds on RSL3-induced cytotoxicity in HK2 and MPC5 cells. As shown in Figure 2c, none of these analogues offered protection against ferroptosis induced by RSL3 in HK2 and MPC5 cells. These results support the hypothesis that the furan ring at c-3/4, the benzofuranyl group at c-3/4 and the isopentenyl group at c-6 are required for GC to exert anti-ferroptotic activity.

### 2.3. GC Prevents RSL3-Induced Iron Dysmetabolism in HK2 and MPC5 Cells

**Figure 3:**
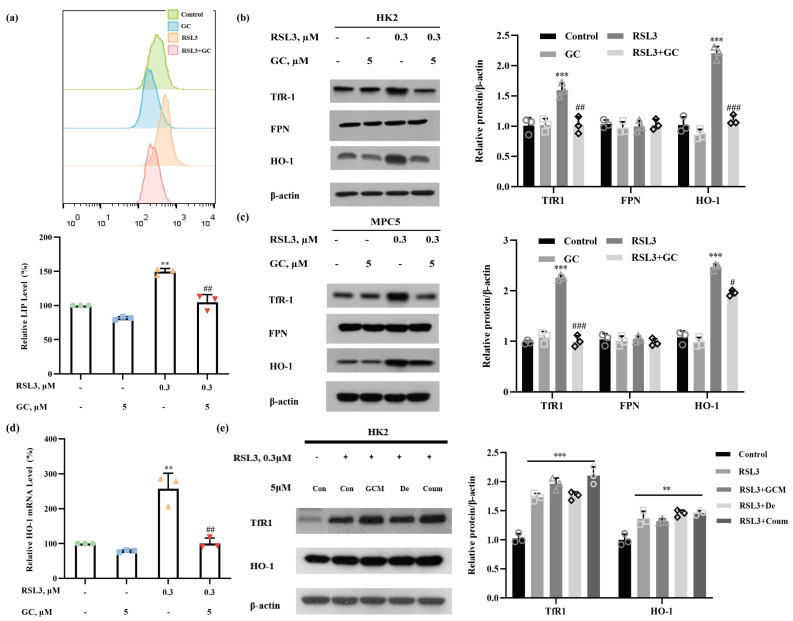
Glycyrol prevented RSL3-induced iron accumulation in vitro. (**a**) The changes in the labile iron pool (LIP) were analyzed using flow cytometry after staining with FerroOrange in HK2 cells; (**b**,**c**) TfR1, HO-1 and FPN protein levels in HK2, MPC5 cells with or without treatment with RSL3 or GC for 18 h; (**d**) The levels of HO-1 mRNA in HK2 cells were evaluated using real-time PCR; (**e**) TfR1 and HO-1 protein levels in HK2 cells with or without treatment with RSL3 or GC chemical structures for 18 h. Data are represented as the means ± SD of triplicate experiments. (** *p* < 0.01, *** *p* < 0.001 versus the control group. # *p* < 0.05, ## *p* < 0.01, ### *p* < 0.001 versus the RSL3 group).

The accumulation of labile iron is one of the main biochemical changes associated with ferroptosis. To elucidate the mechanisms involved in GC-mediated protection against ferroptosis, we examined the changes in the intracellular labile iron pool (LIP) that occurred in response to RSL3, GC or a combination of the two compounds to ascertain whether the protective effect of GC was associated with its ability to regulate iron metabolism. As shown in Figure 3a, the Fe^2+^ level was increased significantly in RSL3-treated HK2 cells; this increase was dramatically attenuated in the presence of GC. To further elucidate the potential targets through which GC regulates iron metabolism, we then measured the changes in the levels of iron-metabolism-related proteins, including transferrin receptor 1 (TfR1), which mediates iron uptake [16]; ferroportin1 (FPN1), which mediates iron export [17]; and HO-1, which catalyzes the degradation of heme to ferrous iron [18]. As shown in Figure 3b, treatment with RSL3 caused a significant increase in the levels of TfR1 and HO-1 without affecting FPN1 in HK2 cells, suggesting that the elevated cellular uptake of iron and the release of iron from heme might contribute to RSL3-induced LIP. Similar results were also found in MPC5 cells (Figure 3c). The increase in the levels of TfR1 and HO-1 proteins following RSL3 treatment was significantly reduced by GC (Figure 3b,c). Furthermore, RT-PCR analysis showed that level of HO-1 mRNA increased in response to RSL3 treatment and that this increase was repressed by GC (Figure 3d), indicating that a transcriptional mechanism might be involved in GC-mediated suppression of HO-1 expression. In addition, the three analogues GCM, De and Coum failed to suppress RSL3-induced changes in TfR1 and HO-1 levels (Figure 3e), a finding consistent with the hypothesis that these analogues could not offer protection against RSL3-induced ferroptosis.

### 2.4. Induction of HO-1 Promotes RSL3-Induced Ferroptosis in HK2 Cells

**Figure 4:**
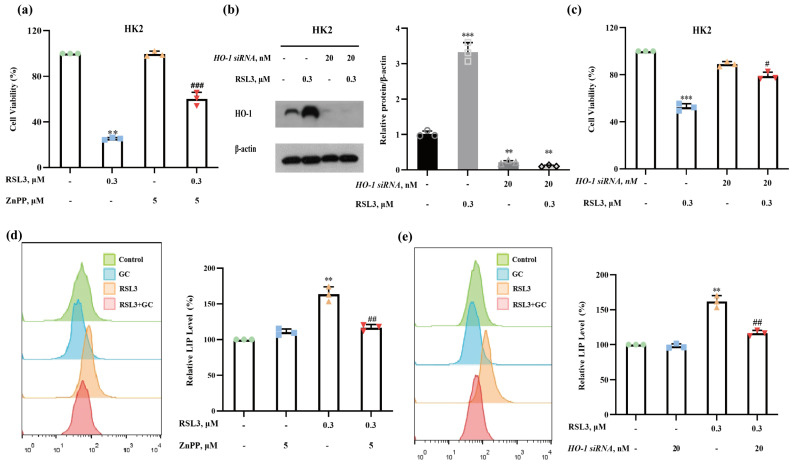
Glycyrol regulated HO-1, thus preventing iron accumulation in vitro. (**a**) HK2 cells were treated with RSL3 (0.3 μM) in the absence or presence of different concentrations of ZnPP for 18 h, and then cell viability was assayed; (**b**) The expression of HO-1 in HK2 cells after transfection with HO-1 siRNA or negative-control siRNA; (**c**) Effects of HO-1 knockdown on cell viability; (**d**) The changes in the labile iron pool (LIP) in HK2 cells in the absence or presence of ZnPP; (**e**) The changes in the labile iron pool (LIP) in HK2 cells after transfection with HO-1 siRNA or negative-control siRNA. Data are represented as the means ± SD of triplicate experiments. (** *p* < 0.01, *** *p* < 0.001 versus the control group. # *p* < 0.05, ## *p* < 0.01, ### *p* < 0.001 versus the RSL3 group).

It has been shown that HO-1 can exert either pro- or anti-ferroptotic activity, depending on the context. To determine the functional role of HO-1 in RSL3-induced ferroptosis, we evaluated the influences of HO-1 inhibition by its pharmacological inhibitor zinc protoporphyrin (ZnPP) on ferroptosis; the results are shown in Figure 4a. RSL3-induced reduction of cell viability in HK2 cells was significantly ameliorated in the presence of ZnPP, suggesting that the induction of HO-1 by RSL3 facilitated ferroptosis induction in HK2 cells. The promoting effect of HO-1 on RSL3-induced ferroptosis in HK2 cells was further confirmed by a genetic approach. As shown in Figure 4b, knockdown of HO-1 by its specific siRNA led to a significant decrease in HO-1 expression. Under these conditions, the reduction of cell viability by RSL3 was significantly diminished (Figure 4c), offering further supporting evidence for the pro-ferroptotic role of HO-1 in RSL3-induced cytotoxicity in HK2 cells. In line with the pro-ferroptotic effect of HO-1 in response to RSL3, inhibition of HO-1 by either its pharmacological inhibitor or the siRNA approach resulted in a significant reduction in RSL3-induced iron accumulation (Figure 4d,e). These results suggest that the suppression of HO-1 induction by GC contributed to its protective effect against RSL3-induced ferroptosis via repression of HO-1-mediated heme degradation and free-iron release.

### 2.5. GC Suppresses ROS Generation and Lipid Peroxidation

**Figure 5:**
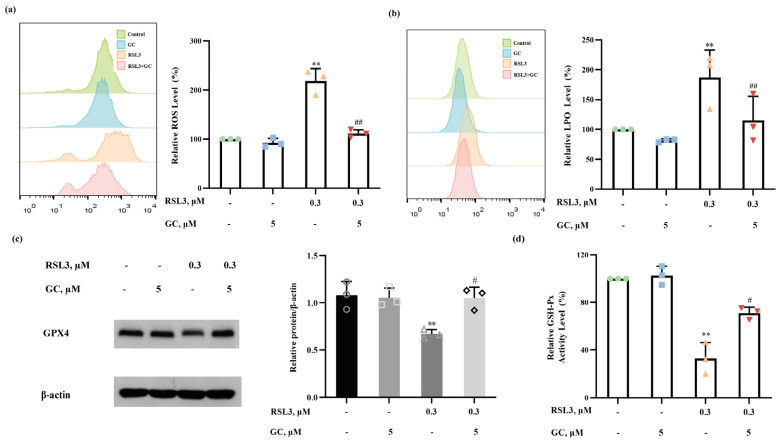
Glycyrol suppressed ROS and lipid peroxidation. After the treatments, the changes in the levels of ROS (**a**) and LPO (**b**) were analyzed using flow cytometry after staining with DCFH-DA and Liperfluo, respectively; (**c**) The expression of GSH peroxidase 4 (GPX4) in each group in HK2 cells; (**d**) GSH-PX activity in each group in HK2 cells. Data are represented as the means ± SD of triplicate experiments. (** *p* < 0.01 versus the control group. # *p* < 0.05, ## *p* < 0.01 versus the RSL3 group).

Oxidative stress and lipid peroxidation play essential roles in ferroptosis. We next investigated the changes in intracellular levels of ROS and lipid peroxides (LPO) in response to RSL3, GC or the combination of the two compounds. As shown in Figure 5a, ROS levels markedly increased with RSL3 treatment, but this effect almost vanished in the presence of GC. As further evidence of the inhibition of oxidative stress, LPO measurement by a Liperfluo probe showed that GC substantially suppressed RSL3-induced LPO generation (Figure 5b). GPX4 is a GSH-dependent enzyme that belongs to the glutathione peroxidase family and is considered to a target through which RSL3 triggers ferroptosis [15,19]. The changes in GPX4 expression were analyzed by western blot. As shown in Figure 5c, the expression of GPX4 was downregulated following RSL3 treatment, while GC significantly inhibited these changes. We also detected GSH-Px activity, and the results are shown in Figure 5d. GC partially reversed the decline in GSH-Px activity caused by RSL3. These findings support the hypothesis that GC protects HK2 and MPC5 cells from RSL3-induced ferroptosis by suppressing ROS and lipid peroxidation.

### 2.6. GC Protects against Folic Acid-Induced Acute Kidney Injury In Vivo

**Figure 6:**
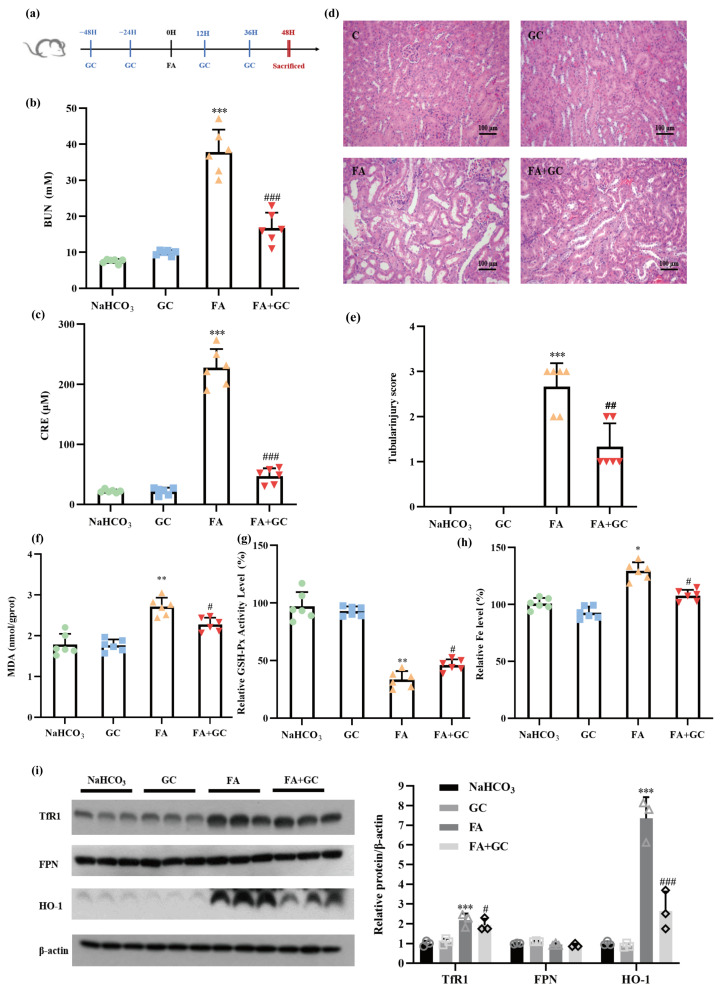
Glycyrol protected against folic acid-induced acute kidney injury in vivo. (**a**) Schematic diagram of the experimental design, showing mice that were treated with FA or pretreated with GC. The levels of blood urea nitrogen (BUN) (**b**) and serum creatinine (**c**) among the different groups. Group NaHCO_3_, mice treated with vehicle; group GC, mice treated with GC alone; group FA, mice treated with FA alone; group FA+GC, mice treated with FA plus GC. Values are presented as the means ± SD (*n* = 6); (**d**) Image of kidney H&E staining of each group, 200×; (**e**) Score of renal-tubule injury of each group; (**f**) MDA level in the kidney in each group; (**g**) GSH-Px activity in the kidney in each group; (**h**) Fe level in the kidney in each group; (**i**) The expression levels of transferrin TfR1, HO-1 and FPN in kidney tissue lysates, as measured using western blotting. Data are represented as means ± SD (*n* = 6). (* *p* < 0.05, ** *p* < 0.01, *** *p* < 0.001 versus the NaHCO_3_ group. # *p* < 0.05, ## *p* < 0.01, ### *p* < 0.001 versus the FA group).

Having found a protective effect of GC against renal-cell ferroptosis in vitro, we next asked whether the protective effect could be achieved in vivo. Folic acid (FA)-induced AKI has been suggested to be a suitable model for recapitulating human kidney-disease phenotypes, to which ferroptosis contributes substantially [20]. Therefore, FA-induced AKI was employed to validate the findings described above in vitro. A brief summary of the experimental design is shown in Figure 6a. Biochemical analysis showed that treatment with FA for 48 h caused a significant increase in levels of blood urea nitrogen (BUN) and creatinine (CRE), which are the key kidney-injury biomarkers, indicating that acute kidney injury was induced by FA, whereas co-treatment with FA and GC dramatically repressed the increase in the levels of BUN and CRE (Figure 6b,c). Histopathological analysis by H&E staining revealed obvious dilatation of the renal tubule and infiltration of inflammatory cells in the renal interstitium and showed that these changes were significantly attenuated by GC, whereas GC alone did not cause any pathological changes (Figure 6d,e). These results suggest that administration of GC could significantly improve the pathological features of acute renal injury in an FA-induced AKI model.

To further explore whether GC’s protection against AKI was associated with its inhibitory effect on nephrocyte ferroptosis, we evaluated the changes in ferroptosis-related markers in kidney samples. As shown in Figure 6f–h, FA caused a significant increase in MDA levels and a decrease in GSH-Px activity, along with an increase in iron content, whereas GC reduced the MDA content, improved the level of GSH-Px activity and decreased the iron accumulation in kidney tissues in the FA-induced AKI model. As was found in the cell-culture experiments, FA significantly increased the expression levels of TfR1 and HO-1 but had no significant effect on the expression of FPN. Such changes, especially the induction of HO-1 expression by FA, were dramatically suppressed by GC (Figure 6i). The results indicate that GC could effectively protect the kidneys from FA-induced damage that is mechanistically associated with regulation of iron homeostasis.

## 3. Discussion

As discussed above, a growing number of studies support the involvement of ferroptosis in certain types of AKI; therefore, a ferroptosis-based approach is a reasonable strategy for managing AKI. Indeed, a number of phytochemicals have been identified to be effective against AKI via repression of ferroptosis [21,22]. In the present study, we investigated the protective effects of GC, a representative coumarin compound found in licorice, against nephrocyte ferroptosis and the related acute kidney injury using both in vitro and in vivo models. We found that GC significantly inhibited nephrocyte ferroptosis induced by RSL3, a well-known ferroptosis inducer, and effectively attenuated folic acid-induced ferroptosis-mediated kidney injury in a mouse model. The findings identified GC as a novel phytochemical for altering the course of nephrocyte ferroptosis.

Disruption of iron homeostasis is a hallmark of ferroptosis. Multiple proteins have been found to be involved in regulating iron metabolism, including TfR1 and HO-1 [16,23]. Regarding the changes in iron metabolism in response to RSL3, our data showed that exposure of HK2 cells to RSL3 resulted in an increase in intracellular labile iron concentrations, indicating that the iron homeostasis was impaired by RSL3. Further analysis of iron-metabolism-related proteins revealed that iron dyshomeostasis by RSL3 was associated with up-regulation of TfR1 and HO-1. As discussed above, heme is a substrate of HO-1, and the induction of HO-1 is believed to promote heme degradation and release of free iron. Therefore, it is reasonable to suggest that the activation of HO-1 promotes iron-dependent ferroptosis. Indeed, some studies have shown that HO-1 can exert a promoting effect on ferroptosis. For example, a study by Fang et al. [24] reported that the up-regulation of HO-1 played a critical role in doxorubicin-induced ferroptosis in cardiomyocytes, an effect that was attributed to its ability to degrade heme, which leads to free-iron release and membrane-lipid peroxidation. HO-1-mediated ferroptosis has been also demonstrated in retinal pigment epithelial cells [25]. In addition, the pro-ferroptotic effect of HO-1 has been also found in erastin-induced ferroptosis in cancer cells [26,27]. In contrast, some other reports support a protective effect of HO-1 against ferroptosis. For instance, a study by Guerrero-Hue et al. demonstrated that curcumin inhibited myoglobin (Mb)-induced ferroptosis in renal tubular cells via activation of cytoprotective HO-1 [28]. Also, the protective effect of HO-1 against ferroptosis has been found in the case of ferroptosis induced by either erastin or RSL3 in murine renal proximal-tubule cells [29]. Therefore, the functional role of HO-1 in regulating ferroptosis is complex and context-dependent. One possible explanation for the lack of agreement surrounding the role of HO-1 is that HO-1 activates both cytoprotective and pro-ferroptotic signaling simultaneously and that the net effect of HO-1 on ferroptosis depends on which signal is stronger, a factor that varies with types of cells and types of ferroptosis inducers. The mechanistic determinants that govern the distinct role of HO-1 in regulating ferroptosis need to be further investigated. To determine the functional role of HO-1 in RSL3-induced ferroptosis in HK2 cells, either a pharmacological inhibitor of RSL3 or a genetic approach was used to inactivate HO-1. This step was followed by measuring the changes in ferroptosis in response to RSL3, and the results showed that the inhibition of HO-1 by both approaches offered significant protection against RSL3-induced ferroptosis in HK2 cells, offering supporting evidence for a promoting role of HO-1 in RSL3-induced ferroptosis in the current experimental setting. This finding was the opposite of that found in a model of RSL3-induced ferroptosis in murine renal cells [29]. The lack of agreement regarding the role of HO-1 in RSL3-induced ferroptosis in murine and human renal cells might be associated with concentrations used in these two studies (0.3 μM in the present study vs. 10 μM in the previous study). We speculated that the HO-1-mediated cytoprotective effect against ferroptosis is stronger than its pro-ferroptotic effect when the cells were exposed to high concentrations of RSL3. Conversely, the pro-ferroptotic effect of HO-1 is stronger than its cytoprotective effect at low concentrations of RSL3. This hypothesis needs to be tested in follow-up studies. Furthermore, we found that the increase in HO-1 expression associated with RSL3 was significantly reduced by GC in vitro and in vivo and that this effect was well correlated with the suppression of ferroptosis. As far as the mechanisms by which GC repressed HO-1 expression are concerned, we demonstrated the involvement of transcriptional inhibition of HO-1 by GC, an effect that was speculated to be associated with regulation of HO-1’s transcriptional factors, Nrf2 and Bach-1, or with regulation of microRNAs. The findings provide mechanistic evidence to support GC-mediated protection against nephrocyte ferroptosis.

In summary, we provide evidence that GC is able to inhibit renal-cell ferroptosis and ameliorate ferroptosis-mediated AKI, an effect mechanistically associated with prevention of iron accumulation and suppression of lipid peroxidation. The findings suggest that GC holds potential for development as a novel agent for treating ferroptosis-related AKI.

## 4. Materials and Methods

### 4.1. Chemicals and Reagents

Glycyrol (GC) and glycycoumarin (GCM) were obtained from BioBioPha (Kunming, China). Demethylsuberosin (De), coumestrol (Coum), RSL3, deferoxamine mesylate (DFO), ferrostatin-1 (Fer-1), liproxstatin-1 (Lip-1) and zinc protoporphyrin (ZnPP) were purchased from MedChem Express (Monmouth Junction, NJ, USA). Folic acid (FA) was purchased from Aladdin (Shanghai, China). 2′,7′-dichlorodihydrofluorescein diacetate (DCFH-DA) was purchased from Sigma Chemical Co. (St. Louis, MO, USA). FerroOrange and Liperfluo were purchased from Dojindo (Kyushu, Japan). Antibodies specific for FTH1, TfR1, FPN, HO-1 and β-actin were purchased from Cell Signaling Technology (Beverly, MA, USA). The secondary antibodies horseradish peroxidase-linked goat anti-rabbit IgG and horseradish peroxidase-linked goat anti-mouse IgG were obtained from MBL Beijing Biotech Co., Ltd. (Beijing, China). A HiFiScript gDNA Removal cDNA Synthesis Kit (CW2582M) and UltraSYBR Mixture (CW0957M) were purchased from Kangwei Century Company (Taizhou, China).

### 4.2. Cell Culture and Treatments

The human proximal tubular epithelial cell line HK2 was obtained from the American Type Culture Collection (ATCC) and grown in Dulbecco’s modified Eagle’s medium (DMEM) supplemented with 10% fetal bovine serum (FBS). The mice podocyte cell line MPC5 was obtained from ATCC and grown in RPMI 1640 supplemented with 10% FBS. Cells were cultured at 37 °C in a humidified atmosphere of 5% CO_2_. Cells were treated with different concentrations of GC, RSL3 and/or other agents for the time indicated in complete medium.

### 4.3. Crystal Violet Staining

Cells (1 × 10^5^ cells/well) were seeded in a 12-well plate. After treatment with GC, RSL3 or other agents for the times indicated, the culture medium was aspirated and replaced with 1% glutaraldehyde solution for 15 min. Then, the cells were stained with a 0.02% crystal violet solution for 30 min. After that, the solution was replaced by 70% ethanol for solubilization. The OD value at 570 nm was measured by microplate reader.

### 4.4. Labile-Iron-Pool Assay

Cells (2 × 10^5^ cells/well) were seeded in a 6-well plate. After the treatments, the cells were trypsinized and washed with PBS and then incubated with 1 μM FerroOrange. After incubation for 30 min at 37 °C in the dark, the cells were used for fluorescence analysis with a Becton Dickinson flow cytometer. The excitation wavelength and emission wavelength were 535 nm and 585 nm, respectively. MFI data were obtained through Flowjo software version 10.0.7. Then, SPSS version 24.0 software was used to analyze the flow-cytometry results and to analyze whether there were significant differences between the groups. Finally, the results are shown in the form of bar graphs produced by GraphPad Prism 8.0.

### 4.5. Measurement of Reactive Oxygen Species (ROS)

Cells (2 × 10^5^ cells/well) were seeded in a 6-well plate. After the treatments, the cells were trypsinized and washed with PBS and then incubated with 20 μM DCFH-DA. After incubation for 30 min at 37 °C in the dark, the cells were washed with PBS three times and resuspended in the PBS, then subjected to fluorescence analysis with a Becton Dickinson flow cytometer. The data-analysis process is described in Section 4.4.

### 4.6. Detection of Lipid Peroxide

Cells (2 × 10^5^ cells/well) were seeded in a 6-well plate. After the treatments, the cells were trypsinized, washed with PBS and then incubated with 1 μM Liperfluo. After incubation for 30 min at 37 °C in the dark, the cells were used for fluorescence analysis with a Becton Dickinson flow cytometer. The excitation wavelength and emission wavelength were 488 nm and 535 nm, respectively. The data-analysis process is described in Section 4.4.

### 4.7. RNA Interference

The HK2 cells were transfected with 20 nM of HO-1-siRNA or negative-control siRNA using INTERFERin siRNA (Santa Cruz, CA, USA) transfection reagent according to the manufacturer’s instructions. At 24 h post-transfection, the cells were used for subsequent experiments.

### 4.8. Quantitative Polymerase Chain Reaction (PCR)

Total RNA from HK2 cells was extracted using the TRIzol method and converted to complementary DNA using HiFiScript gDNARemoval cDNA Synthesis Kit (Kangwei Century Company, Taizhou, China). qPCR was performed using UltraSYBR Mixture according to the manufacturer’s instructions in a thermocycler (MyCycler, Bio-Rad, Hercules, CA, USA). Results were analyzed using the 2^−ΔΔCt^ method.

### 4.9. Animal Experiments

6–8-week-old male Balb/c mice were purchased from Charles River Laboratory (Beijing, China). Animals were allowed to acclimatize for a week under specific pathogen-free conditions at 22 ± 2 °C with 55 ± 10% relative humidity and 12-h day/light cycles and were supplied with standard laboratory chow and water. All experiments were performed in accordance with the guidelines established in the Principles of China Agricultural University Institutional Animal Care and Use Committee. The mice were randomly divided into four groups: control group (0.3 M NaHCO_3_ aqueous solution), GC (10 mg/kg bw/i.p.), FA (250 mg/kg bw, dissolved in 0.3 M NaHCO_3_ aqueous solution/i.p.) and GC+FA (each group: *n* = 6). The dose of GC was determined according to our previous study [14] and dose-finding experiments. The mice were sacrificed at 48 h after the FA administration (a schematic diagram of the experimental design is shown in Figure 6a). The treatments for each group are shown in Table 1. Blood samples were obtained from the abdominal aorta for further experiments. One kidney was fixed in 4% phosphate-buffered formaldehyde for histological analyses, and the other one was frozen for subsequent molecular analysis.

### 4.10. Biochemical Serum and Tissue Analysis

The blood urea nitrogen (BUN), serum levels of creatinine (CRE) and MDA, kidney GSH-Px activity and kidney iron levels were measured by using corresponding reagent kits (BUN kit: #Cat. C013-2-1; CRE kit: #Cat. C011-2-1; MDA kit: #Cat. A003-1-2; GSH-Px kit: #Cat. A005-1-2; Tissue Iron assay kit: #Cat. A039-2-1) from the Nanjing Jiancheng Bioengineering Institute (Nanjing, China) according to the manufacturer’s instructions.

### 4.11. Haematoxylin and Eosin Staining

Renal tissues were fixed with 4% paraformaldehyde overnight, embedded in paraffin, sectioned (4 μm) and stained with haematoxylin and eosin (H&E) according to the manufacturer’s protocol. Damage to the renal tubule was estimated as described previously [22].

### 4.12. Western Blotting

The cells and mouse renal tissues were lysed in RIPA buffer with a protease-inhibitor cocktail. Denatured protein extracts were resolved by electrophoresis and transferred to a polyvinylidene fluoride (PVDF) membrane (0.2 μm). After blocking with 5% non-fat dry milk in TBST buffer, the membranes were probed with primary antibodies overnight at 4 °C. Afterwards, the membranes were probed with HRP-conjugated secondary antibody for 1 h at room temperature. Enhanced chemiluminescence was used to visualize the changes in protein expression. Image J version 1.8.0 software was used to analyze the relative expression levels.

### 4.13. Statistical Analysis

Data are presented as the means ± SD. These data were analyzed by ANOVA with appropriate post hoc comparisons among means using SPSS version 24.0 software. A value of *p* < 0.05 was considered statistically significant.

## Figures and Tables

**Table 1:** List of animal groups with the different treatments.

Groups	Treatments
NaHCO_3_	Intraperitoneal injection, equivalent volume of Tween-80: PBS (5:95, *v*/*v*) solution, equivalent volume of 0.3 M NaHCO_3_ aqueous solution
GC	Intraperitoneal injection, GC 10 mg/kg bw, equivalent volume of 0.3 M NaHCO_3_ aqueous solution
FA	Intraperitoneal injection, equivalent volume of Tween-80: PBS (5:95, *v*/*v*) solution, FA 250 mg/kg bw
FA+GC	Intraperitoneal injection, GC 10 mg/kg bw, FA 250 mg/kg bw

## Data Availability

Data is contained within the article.

## Abbreviations

AKI: acute kidney injury; BUN, blood urea nitrogen; Coum, Coumestrol; CRE, creatinine; De, Demethylsuberosin; DFO, deferoxamine mesylate; FA, folic acid; Fer-1, ferrostatin-1; FPN, ferroportin1; GC, glycyrol; GCM, Glycycoumarin; GPX4, GSH peroxidase 4; HO-1, Heme Oxygenase-1; Lip-1, liproxstatin-1; LIP, labile iron pool; LPO, lipid peroxides; MDA, malondialdehyde; TfR1, transferrin receptor 1; ROS, reactive oxygen species; ZnPP, zinc protoporphyrin.
